# Overexpression of *Cymbidium goeringii* Cgo-miR159 Regulates Anther Dehiscence and Pollen Development in *Arabidopsis* and Tobacco

**DOI:** 10.3390/genes16010035

**Published:** 2024-12-29

**Authors:** Zihan Xu, Qian Liu, Yue Chen, Jinming Wang, Jianshuang Shen, Fengrong Hu

**Affiliations:** 1 Animation & Game College, Hangzhou Vocation & Technical College, Hangzhou 310018, China; 2024010048@hzvtc.edu.cn; 2 College of Landscape Architecture, Nanjing Forestry University, Nanjing 210037, China; 3 Institute of Horticulture, Zhejiang Academy of Agricultural Science, Hangzhou 310021, China; 4 Faculty of Science, University of Hong Kong, Hong Kong 999077, China

**Keywords:** miR159, *Cymbidium*, anther dehiscence, pollen development

## Abstract

Background: MicroRNA159 (miR159) is a conserved miRNA found in various plant species. By regulating GAMYB-like transcription factors, miR159 is involved in diverse biological processes. *Cymbidium goeringii*, a significant traditional Chinese orchid, has unique flower shape and elegant fragrance. However, its development has been several limited because of the low flower bud differentiation and the difficult reproduction. This research aims to provide guidance for the role of cgo-miR159 in reproductive organ development to enhance the ornamental and economic value of *Cymbidium*. Methods: In this study, miR159 was cloned and its expression was determined across different development stages and tissue types. The function of cgo-miR159 was identified using gene transformation in *Arabidopsis* and tobacco plants. Results: High expression levels of cgo-miR159 were detected in the leaves and stamens during reproductive growth and expression peaked during flower bud development when the flower was above 0.5 to 3 cm in length. In transgenic experiments, the ectopic expression of cgo-miR159 led to defective development in the stamens of model plants (*Arabidopsis* and tobacco), including earlier anther dehiscence and pollen deformity, which resulted in developmental abnormalities and reduced seeds count in fruits. Conclusions: In summary, cgo-miR159 affected the development of reproductive organs in model plants. This research complements previous studies on the function of miR159 and provide useful references for the genetic improvement of orchids.

## 1. Introduction

*C. goeringii* is a terrestrial orchid belonging to the Orchidaceae family with a long cultivation history in China. It is known as the “spring orchid” because it blooms from later January to early March. Due to its diverse floral morphological patterns and colors, *C. goeringii* has great ornamental and economic value [1]. However, wild seedlings of *C. goeringii* have a long breeding cycle, difficult reproduction, and low flower bud differentiation, which restrict the development of its industry. Therefore, an exploration of the mechanisms related to the growth and development of *C. goeringii* is important for artificial breeding programs.

MicroRNAs (miRNAs) are a class of endogenous non-coding small RNA molecules that play flexible regulatory roles in a wide variety of biological processes during the major growth and developmental stages of plants [2]. Current studies on the effects of miRNAs have mainly focused on crops and model plants, laying a solid foundation for exploring their mechanisms in ornamental plants. The miR159 is a highly conserved miRNA that is a key regulator of the development of reproductive organs [3,4]. Thus, mastering the regulatory mechanism of miR159 is helpful to understand the reproductive development process of plants. At present, there have been some reports of miR159 occurrence in orchids, such as in *Phalaenopsis* [5] and *Oncidium* [6], but its specific function and regulatory mechanisms remain unclear.

miR159 can affect the regulation of flowering time in plants via interaction with the MYB transcription factor [7]. The miR159–*MYB* pathway is conserved in diverse plants and functions through gibberellin (GA)-mediated signal transduction. For example, the overexpression of miR159 delays flowering in *Gloxinia* (*Sinningia speciosa*) and the *Landsberg erecta* (*Ler*) laboratory strain of *Arabidopsis thaliana* [8,9]. *GAMYB*, a gene in the gibberellin signal pathway, is involved in this process. This gene and its homolog gene (*GYMYB*-like) are the only conserved targets of miR159 [10]. In addition, miR159 affects vegetative growth in *Arabidopsis* by altering the expression of miR156, thereby shifting flowering time. When miR159 was absent, its target gene *MYB33* promoted the transcription of *MIR156A*, *MIR156C,* and their target *SPL9*, by directly binding to the promoters of these three genes, thus changing the vegetative phase and delaying flowering. Conversely, the overexpression of miR159 has been observed to result in early flowering in *Arabidopsis* [11], which differs from the results of other studies.

Anther development is a crucial biological process in the sexual reproduction of plants. Abnormal expression of miR159–*MYB* can lead to abnormal anther development and male sterility and is conserved across various plant species, such as *Arabidopsis*, rice, wheat, cucumber, and *Brassica campestris* [12,13,14,15]. The tapetum undergoes hypertrophy at the pollen mother cell stage in *myb33 myb65 Arabidopsis*, resulting in pre-meiotic abortion [16,17]. GA functions in the anther to promote exine and Ubisch body formation by *GAMYB*-dependent induction of *CYP703A3* in rice [18]. Overexpressed *Bra-MIR159a* contributes to promoting pollen shrinkage and abnormal pollen germination [19]. These results provide guidance for our study in orchids.

miR159 also plays a critical role in the development of fruit and seeds. The miR159–*GAMYB1*/*2* module is crucial for fruit set in tomatoes. Overexpression of *SlMIR159* in tomato resulted in abnormal ovule development, which led to the formation of seedless fruits. *SlGAMYB1*/*2* silencing in *SlMIR159*-overexpressing plants resulted in the misregulation of pathways associated with ovule and female gametophyte development [20]. In *Arabidopsis*, miR159a or miR159b single mutants show no phenotypic changes compared to the wild-type *Arabidopsis*, whereas miR159ab double mutants exhibit shorter fruits and smaller seeds [17]. When *GAMYB* was inhibited by RNAi in strawberries (*Fragaria* × *Ananassa*), the time and color of fruit ripening changed [21].

In this study, a flowering-associated miRNA of *C. goeringii*, miR159, was overexpressed in *Arabidopsis* and tobacco. Since this miR159 was highly expressed in the stamens, it was hypothesized that it might influence the development of reproductive organs or other biological processes. Based on these considerations, this study further investigated the effects of cgo-miR159 on the development of the anthers, pollen, stigma, and other reproductive organs in *Arabidopsis* and tobacco. These results contribute to a basic understanding of the miRNA regulatory mechanisms of growth and development in *C. goeringii*, and provide a direction for future research on molecular breeding in ornamental orchids.

## 2. Materials and Methods

### 2.1. Plant Materials

*C. goeringii* cultivar Songmei, tobacco (*Nicotiana tabacum*), and *Arabidopsis thaliana Col-0* ecotypes were used in this study.

The plant tissues of *C. goeringii* were collected from 2-year-old ‘Songmei’ plant. Each pseudobulb was considered a single seedling, and five seedlings were planted together in one pot. These pots were then cultivated on the seedbed in a naturally lit glasshouse at the Institute of Horticulture, Zhejiang Academy of Agricultural Sciences, Hangzhou, China, under the conditions of 70% relative humidity, a temperature of 22 °C, and natural light.

*Arabidopsis* with a Columbia ecotype (Col-0) background was grown at 22 °C under 75% relative humidity, the illumination intensity of 6000–8000 lx, and a 16 h day/8 h night photoperiod in an artificial climate chamber. One plant was planted per pot.

*Nicotiana tabacum* was grown under LD conditions (16 h light/8 h dark, the illumination intensity of 8000–10,000 lx) with 60% humidity in a growth chamber at 22–23 °C. One plant was planted per pot as well.

### 2.2. RNA Extraction and RT-qPCR Analysis

Total small RNA was extracted from different tissues using a MiniBEST Universal RNA Extraction Kit (Takara, Shiga, Japan). Approximately 1 μg of small RNA was used as a template for first-strand cDNA synthesis using the miRNA 1st Strand cDNA Synthesis Kit (Vazyme, Nanjing, China) with a stem-loop primer. RT-qPCR was performed using the miRNA Universal SYBR qPCR Master Mix (Vazyme, Nanjing, China) and a StepOnePlus real-time PCR system (Thermo Fisher, Waltham, MA, USA). The RT-qPCR products were amplified with 1 μL template of RT reaction mixture, 10 μL 2× ChamQ Universal SYBR qPCR Master Mix, 0.4 μL mQ Primer (10 μmol/L), 0.4 μL specific cgo-miR159 primer (10 μmol/L), and ddH_2_O to a final volume of 20 μL. The PCR procedure was 95 °C for 5 min; 40 cycles of 95 °C for 10 s and 60 °C for 30 s; and the dissolution curves were obtained by one cycle of 95 °C for 15 s, 60 °C for 1 min, and 95 °C for 15 s. We used *Actin* as an internal reference gene for *C. goeringii*, *Arabidopsis*, and tobacco. The primers are listed in Supplementary Table S1.

### 2.3. Gene Cloning and Bioinformatic Analysis

The mature miR159 was obtained from sRNA-seq, and then the full-length transcriptome sequencing (sequencing by synthesis, SMRT) was performed. By comparing with the sequencing data and miR159 precursors from other species in miRBase, the miR159 precursor sequence of *C. goeringii* was obtained. The precursor sequence of miR159 (pre-miR159) from *C. goeringii* was amplified using the primer pairs listed in Table S1 and cloned into the *pBI121* vector at double restriction sites (*XbaI* and *SmaI*) to construct the recombinant vector. Gene amplification was performed using a standard PCR procedure of 94 °C for 3 min; 35 cycles of 98 °C for 10 s, 60 °C for 10 s, and 72 °C for 10 s; and 72 °C for 5 min.

Secondary structure prediction was performed using the RNAfold online tool: http://rna.tbi.univie.ac.at (accessed on 18 January 2024). The homologous sequences of the precursor and mature cgo-miR159 were downloaded from miRBase: http://www.mirbase.org (accessed on 18 January 2024) and aligned using DNAMAN 9.0 software. A phylogenetic tree based on the alignment was constructed using the neighbor-joining (NJ) algorithm in MEGA 6.0 [22], and bootstrap values were calculated with 1000 replicates.

### 2.4. Genetic Transformation of Arabidopsis and Tobacco

The overexpression vector *pBI121-35S:MIR159* was transformed into *Arabidopsis Col-0* using *Agrobacterium tumefaciens* strain *GV3101*-mediated floral dipping method [23]. Transgenic seeds were screened on MS medium containing 50 mg/L kanamycin until T3 homozygous plants were obtained and used for further experiments. The seeds of the wild-type were grown and harvested side-by-side with the transgenic lines under the same conditions. Genetic transformation of tobacco was performed according to the leaf-disk genetic transformation system with slight modifications [24]. Tissues from young leaves were infected with *Agrobacterium* strain *EHA105* on a co-cultivation medium for 2 d. After co-cultivation, the leaf explants were transferred to a medium containing kanamycin and cefotaxime to select transgenic adventitious shoots and eradicate *Agrobacterium*. The wild-type leaves were differentiated on medium without kanamycin. Kanamycin-resistant shoots containing cgo-miR159 and WT shoots of similar size were subcultured in the rooting medium without kanamycin for about 3 weeks and then transferred into the soil. Leaf tissue samples were collected from each positive transgenic plant for DNA extraction and PCR analysis (Figure S1a–d). Finally, *Arabidopsis* selected line 5 and 12 for phenotypic observation, and tobacco selected mixed samples of line 2, 3, 5 for phenotypic observation.

### 2.5. Alexander Staining and In Vitro Pollen Germination

Alexander staining of pollen and stamens was performed as previously described [25]. Pollen from tobacco plants was collected from recently opened flowers before anther dehiscence. After the anthers were dried to dehiscence, pollens were extracted by sieving, and stored at 4 °C for use in vitro pollen germination. Pollen was added to a liquid germination medium (10% sucrose, 25 mg/L boric acid, pH 5.8) to achieve a final concentration of 2.5 g/L. Approximately 30 μL of the mixture was dropped onto a single concave microscope slide, which was incubated at 21 °C on a temperature-controlled plate for 3 h. Pollen germination was determined using a light microscope at 40× magnification and the pollen grains were recorded as germinated when the pollen tube was at least twice the length of the pollen grains.

### 2.6. Scanning Electron Microscopy (SEM)

Pollen collected from fully open flowers of *Arabidopsis* and tobacco were attached to the sample stage of a Quanta 200 Scanning Electron Microscope (SEM; FEI, Hillsboro, OR, USA) and the morphology of the sample was observed after the surface underwent a gold spraying treatment. The stamens and pistils were collected from *Arabidopsis* flowers that were about to bloom, which was determined by the flowers just showing their white color. In tobacco, flowers were collected at three stages: buds not opened, buds about to open, and flowers fully open. All stamens and pistils were prepared as described for the pollen used for SEM. Images were obtained at an accelerating voltage of 25 kV.

### 2.7. Anther Dehiscence Observation

Tobacco flower buds were collected during two different periods: buds not opened and about to bloom, and the corolla was removed. Anther dehiscence in wild-type and transgenic plants was observed using a camera at room temperature for 3 h.

### 2.8. Seed Index Determination

The total number of seeds per fruit (NT) and 1000-seed weight were determined. At least 10 fruits were observed in each transgenic line.

### 2.9. Statistical Analysis

IBM SPSS 24.0 statistics software package for Windows and Microsoft Excel (2016) was used to calculate descriptive statistics. The significance of differences was estimated using ANOVA and Duncan’s tests. Diagrams were drawn using the Origin software (2017).

## 3. Results

### 3.1. Identification and Analysis of Cgo-miR159

**Figure 1:**
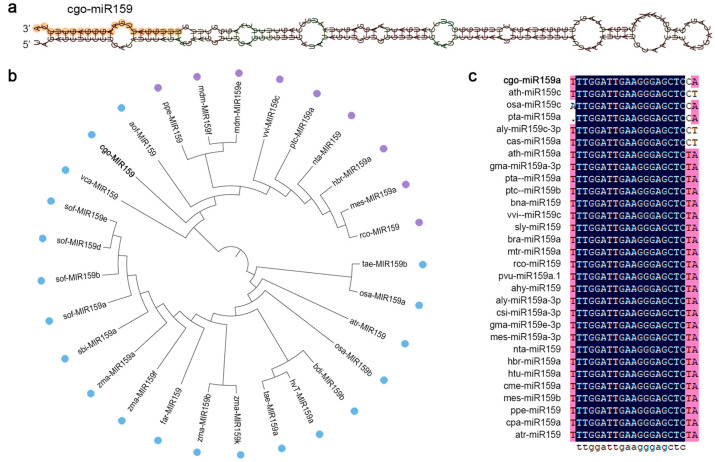
Bioinformatics analyses for miR159 in *C. goeringii*. (**a**) Predicted stem-loop structure of pre-miR159. Mature miRNA sequence is highlighted; (**b**) phylogenetic trees for precursor miR159 sequence showing homology with several published pre-miR159s in miRBase. The blue dots represent monocotyledons and the purple dots represent dicotyledons. (**c**) Nucleotide sequence alignment of mature miR159 sequence with other homologous sequences. Ahy, *Arachis hypogaea*; aly, *Arabidopsis lyrate*; aof, *Asparagus officinalis*; ath, *Arabidopsis thaliana*; atr, *Amborella trichopoda*; bdi, *Brachypodium distachyon*; bna, *Brassica napus*; bra, *Brassica rapa*; cas, *Camelina sativa*; cme, *Cucumis melo*; cpa, *Carica papaya*; csi, *Citrus sinensis*; far, *Festuca arundinacea*; gma, *Glycine max*; hbr, *Hevea brasiliensis*; htu, *Helianthus tuberosus*; hvT, *Hordeum vulgare*; mes, *Manihot esculenta*; mdm, *Malus domestica*; mtr, *Medicago truncatula*; nta, *Nicotiana tabacum*; osa, *Oryza sativa*; ppe, *Prunus persica*; pta, *Pinus taeda*; ptc, *Populus trichocarpa*; pvu, *Phaseolus vulgaris*; rco, *Ricinus communis*; sbi, *Sorghum bicolor*; sly, *Solanum lycopersicum*; sof, *Saccharum officinarum*; tae, *Triticum aestivum*; vca, *Vriesea carinata*; vvi, *Vitis vinifera*; zma, *Zea mays*.

Precursor and mature miR159 sequences in *C. goeringii* were identified based on small RNA sequencing data and cloned from the leaves of the cultivar ‘Songmei’ with lengths of 202 bp and 21 nt, respectively (Table S2). The predicted stem-loop structure of *pre-miR159* is shown in Figure 1a, which had 2 nt overhangs at the 3′ ends of its arms. The minimal folding free energy (MFE) of this structure was −82.20 kcal/mol, the MFE index (MFEI) was 0.93, and there were fewer than five mismatched positions in miRNA:miRNA* duplex, indicating that the sequence is a miRNA homolog [26,27,28].

The *pre-miR159* sequence of *C. goeringii* (*cgo-MIR159*) was retrieved from the miRBase database and the top 28 sequences with high homology were downloaded from 21 species. The phylogenetic tree showed that *aof-MIR159* in *Asparagus officinalis* was closely related to *cgo-MIR159*, followed by *vca-MIR159* of *Vriesea carinata* (Figure 1b). Although these *MIR159* sequences were derived from 20 species, 20 genera, and 10 families, they had a high degree of regularity in their distribution on the tree. The related *MIR159*s were divided into dicotyledonous and monocotyledonous clades, whereas *cgo-MIR159*, *aof-MIR159*, and *vca-MIR159* were located at the junction of these two clades, reflecting the evolutionary relationships of the plant families. In the analysis of mature miRNAs, all homologous sequences differed from cgo-miR159 by only one base (Figure 1c), suggesting that the mature miR159 is relatively conserved among species.

### 3.2. Expression Analysis of Cgo-miR159 in C. goeringii

**Figure 2:**
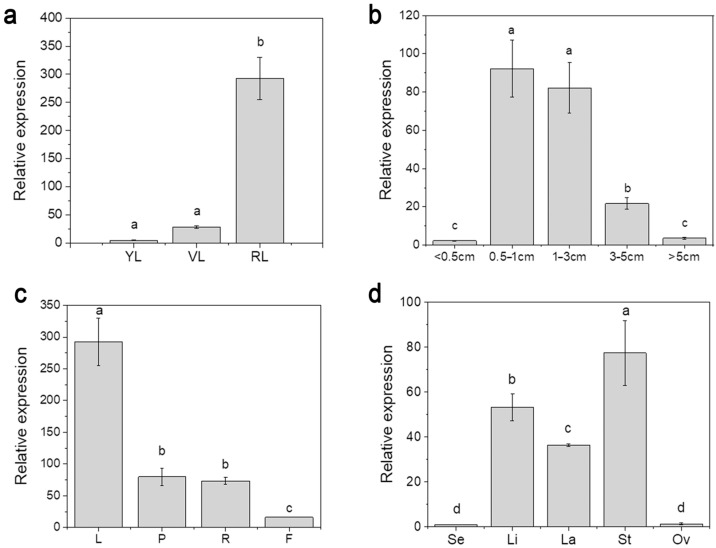
Expression patterns of miR159 in *C. goeringii*. (**a**) Expression level of cgo-miR159 in developing stages of leaves. YL, young leaves; VL, leaves during vegetative growth; RL, leaves during reproductive growth; (**b**) expression level of cgo-miR159 in developing stages of flower buds. The mean lengths of flower buds at the different stages were expressed in cm; (**c**) expression level of cgo-miR159 in various tissues. L, leaves; P, pseudobulbs; R, roots; F, flowers; (**d**) expression level of cgo-miR159 in various floral organs. Se, sepals; Li, Lip petals; La, lateral petals; St, stamens; Ov, ovaries. Bars indicate mean ± SD (*n* = 3). The different letters indicate significant differences (*p* < 0.05).

To analyze the expression pattern of cgo-miR159, its expression at different developmental stages and in various tissues was measured using RT-qPCR. As shown in Figure 2a, miR159 was barely expressed in the leaves of *C. goeringii* during the vegetative growth stages. During flower development, miR159 showed the highest expression when the flower was more than 0.5 cm in length, and then decreased gradually with flower opening (Figure 2b). Four types of tissues were collected during the reproductive growth stage and miR159 was expressed in all examined tissues. The highest expression was observed in the leaves and the lowest was in the flowers (Figure 2c). Finally, among the different flower organs, this miRNA was mainly expressed in the stamens, implying that it may play an important role in stamen development (Figure 2d).

### 3.3. Cgo-miR159 Overexpression Resulted in Abnormal Reproductive Organs in Arabidopsis

**Figure 3:**
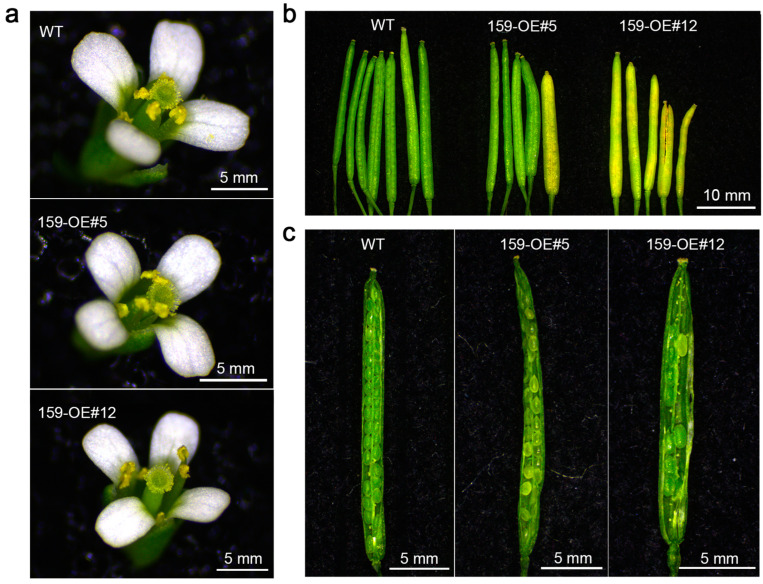
Phenotype changes in flowers and fruit pods of wild-type and cgo-miR159 overexpression *Arabidopsis* under a stereomicroscope; (**a**) compared with the flower characteristics at the same time points, the *35S:cgo-MIR159* plants had a lower amount of pollen attachment; (**b**) compared with fruit morphology, the *35S:cgo-MIR159* fruit pods showed a reduction in fruit size. (**c**) In anatomical images of fruit pods, the number of seeds per fruit in *35S:cgo-miR159* plants was reduced.

Due to the high expression of cgo-miR159 in the stamens of *C. goeringii* and its previous study with male sterility in other plant species, the *35S:cgo-MIR159* construct was transferred into wild-type *Arabidopsis* and two independent transgenic lines from the T_3_ generation were selected for phenotypic observation (Figure S1a,b). Firstly, fully opened flowers of the wild-type and transgenic (overexpressed) lines were observed under a stereomicroscope. The four stamens arranged in the inner whorl of the wild-type were at the same level as the stigma, and a large amount of bright yellow pollen was attached to the anther wall. However, the tetradynamous anthers of *35S:cgo-MIR159* transgenic lines were slightly higher than the stigma and the anthers were dark yellow with less pollen attached, especially in line 12 (Figure 3a). Subsequently, the fruits of each line were randomly selected for anatomical observation (Figure 3b,c). The *35S:cgo-MIR159* overexpressing fruit pod showed a reduction in fruit size and seed number compared to the wild-type (Figure 3b). The mature fruit pods of wild-type *Arabidopsis* had extremely homogeneous widths and similar lengths. The seeds in the pods were close together and showed an ordered arrangement. However, the pods of the transgenic lines turned yellow earlier than those of the wild type, with uneven width and length, and abnormal seed development (Figure 3c)

**Figure 4:**
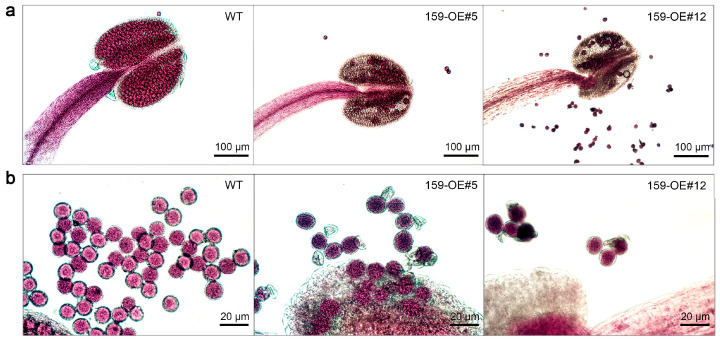
Phenotype changes in stamen and pollen of wild-type and cgo-miR159 overexpression *Arabidopsis* under a light microscope. (**a**) Comparison with the stamen characterization, the anthers of *35S:cgo-MIR159* plants matured and released pollen grains prematurely. (**b**) Comparison with the pollen morphology, the pollen grains of the wild-type were full and round, whereas some pollen grains of *35S:cgo-MIR159 Arabidopsis* were malformed and un-viable.

**Figure 5:**
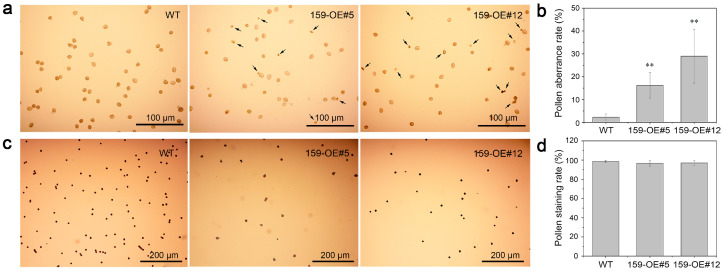
Phenotype changes and statistics in pollen of wild-type and cgo-miR159 overexpression *Arabidopsis* under a light microscope. (**a**) Observation of pollen aberrance rate. Arrows point to the malformed pollen. (**b**) The statistical results of pollen aberrance rate. (**c**) Observation of pollen grains with Alexander solution. (**d**) The statistical results of pollen staining rate. Bars show SD with three technical replicates. Bars indicate mean ± SD (*n* = 20). ** depicts a statistically extremely significant difference (*p* < 0.01).

To further clarify the biological function of cgo-miR159 in stamen development, the anthers and pollen from fully opened flowers were collected for microscopic observation and Alexander staining. At this time, the states of the anthers differed between the wild-type and transgenic plants. The anthers of wild-type plants were full of viable pollen grains; however, in transgenic plants, the anthers matured and released pollen grains prematurely (Figure 4a). The pollen grains of the wild-type were full and round, whereas some pollen grains of transgenic *Arabidopsis* were malformed and unviable (Figure 4b) and the pollen aberrance rate was significantly higher than that of the wild-type (Figure 5a,b). However, there was no significant difference in the Alexander staining rates of mature pollen grains between the two groups (Figure 5c,d).

**Figure 6:**
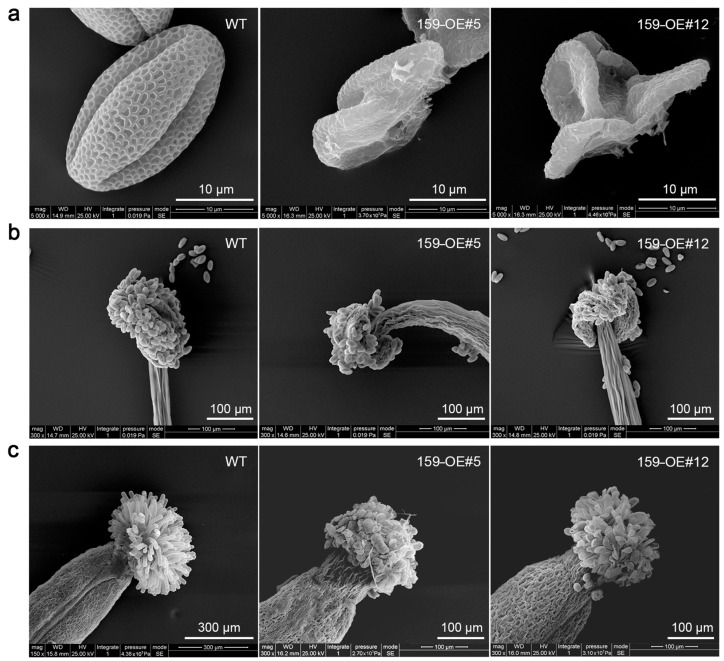
Phenotype changes in (**a**) pollen grains, (**b**) anthers, and (**c**) stigmas of wild-type and cgo-miR159 overexpression *Arabidopsis* under scanning electron microscope (SEM). In *35S:cgo-MIR159* plants, some pollen grains were adhesive and shriveled, while anther maturation and stigma atrophy occurred prematurely.

The SEM results reveal that the pollen grains of the wild-type were full, whereas those of the transgenic lines were adherent and shriveled (Figure 6a). As shown in Figure 6b,c, the anthers maturation and the stigma atrophy was in advance following overexpression of cgo-miR159. All stamens and pistils were collected from flowers that were close to bloom.

### 3.4. Cgo-miR159 Overexpression Affected Stamen and Pistil Development in Tobacco

**Figure 7:**
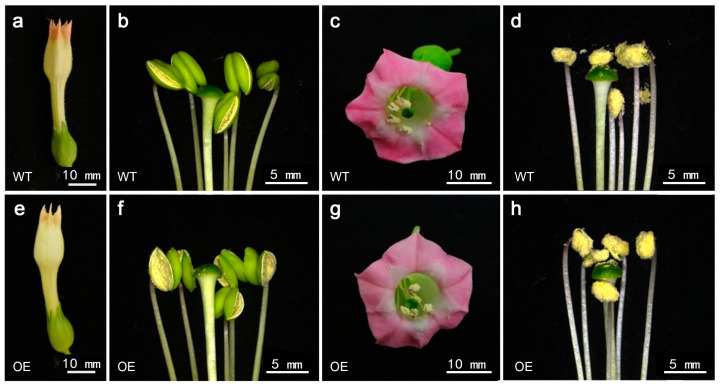
Phenotype changes in floral organs of (**a**–**d**) wild-type and (**e**–**h**) cgo-miR159 overexpression tobacco. The anthers of overexpressed plants were dehiscent in advance, and the morphology of flower organs remained normal. (**a**,**e**) Flowers about to bloom; (**b**,**f**) stamens and pistils collected from flowers about to bloom; (**c**,**g**) fully open flowers; (**d**,**h**) stamens and pistils collected from fully open flowers.

To further confirm the function of cgo-miR159, the *35S:cgo-MIR159* recombinant plasmid was introduced into tobacco plants to investigate whether its overexpression affects flower organ development in tobacco. The flowers that were about to bloom and those that were fully opened in the wild-type and *35S:MIR159* transgenic lines were dissected (Figure 7a–h). The anthers of transgenic flowers dehiscent earlier than those of wild-type flowers and their pollen was clearly attached to the stigma before the flowers opened (Figure 7b,f). Extra-abnormal floral organ development, including floral pattern defects, was not observed in fully open flowers.

**Figure 8:**
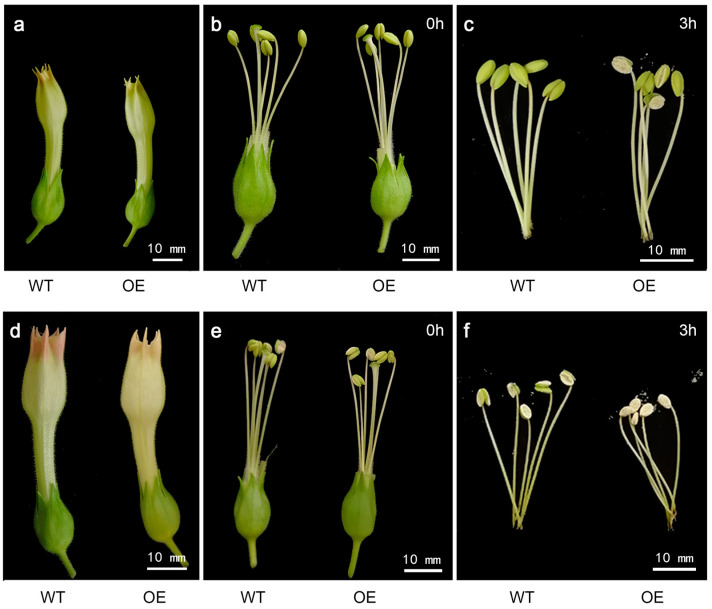
Phenotype changes in anther dehiscence of wild-type and cgo-miR159 overexpression tobacco showed that the anthers of the transgenic plants dehisced earlier, with pollen being released prematurely. (**a**) Flower buds that have not yet shown color; (**b**) stamens collected from flower buds that have not yet shown color; (**c**) stamens collected from flower buds that have not yet shown color after 3 h at room temperature; (**d**) flower buds that were just starting to change color; (**e**) stamens collected from flower buds that have just shown color; (**f**) stamens collected from flower buds that have just shown color after 3 h at room temperature.

To further determine whether ectopic expression of cgo-miR159 advanced the timing of anther dehiscence, flower buds at two stages with the corolla and pistil removed were collected for comparison. After 3 h at room temperature, in vitro anthers of flower buds that did not show color were not dehiscent in the wild-type, but showed early dehiscence in transgenic lines (Figure 8a–c). Similarly, the dehiscence of anthers from colored transgenic flower buds was also faster than those of the wild-type (Figure 8d–f).

**Figure 9:**
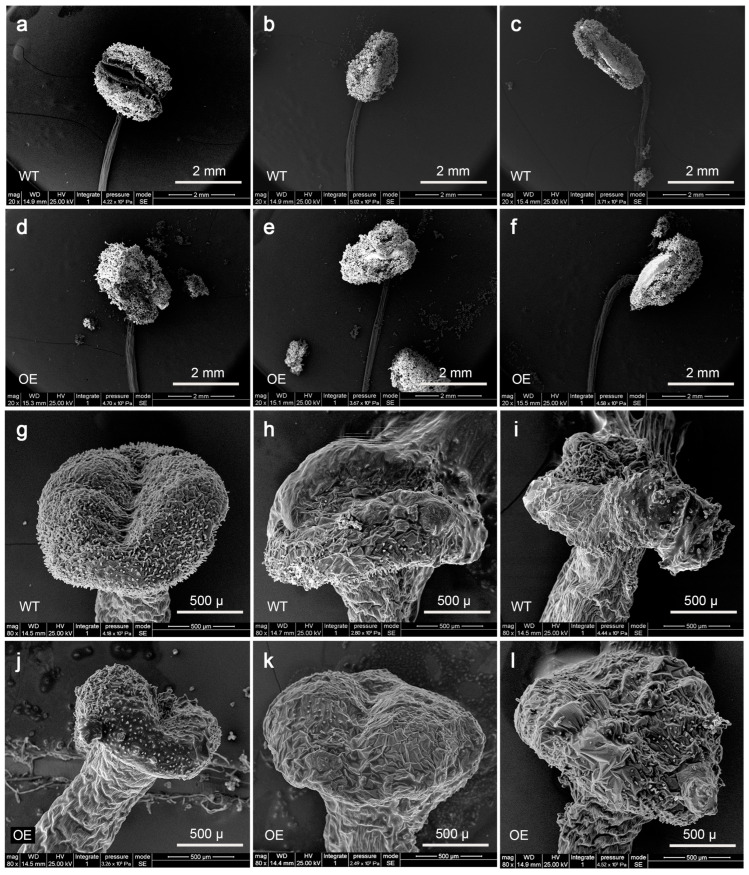
Phenotype changes in anthers and stigmas of wild-type and cgo-miR159-overexpressed tobacco under SEM showed that pollen was released prematurely in the transgenic plants as well, while no significant changes were observed in the stigmas. (**a**,**d**) Anthers collected from flower buds that have not yet shown color; (**b**,**e**) anthers collected from flower buds just starting to change color; (**c**,**f**) anthers collected from fully open flowers; (**g**,**j**) stigmas collected from flower buds that have not yet shown color; (**h**,**k**) stigmas collected from flower buds just starting to change color; (**i**,**l**) stigmas collected from fully open flowers.

Electron microscopy was conducted to observe the anthers and stigmas during each period. The dehiscence of the anthers was almost identical to the results described above. Pollen was released from the transgenic anthers during the first stage, which was earlier than WT (Figure 9a–f). However, no significant differences were observed in the stigmas, especially in papillary cells, between the two genotypes during flowering (Figure 9g–l).

### 3.5. Cgo-miR159 Overexpression Altered Morphology and Viability of Pollen in Tobacco

**Figure 10:**
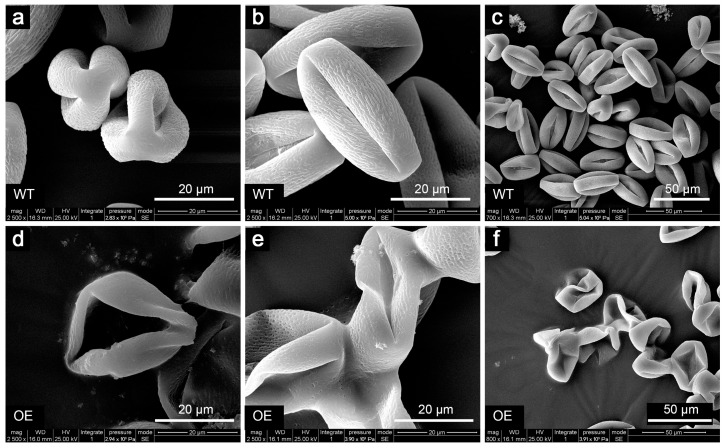
Phenotype changes in pollen morphology of wild-type (**a**–**c**) and cgo-miR159-overexpressed (**d**–**f**) tobacco under SEM show that the pollen grains of transgenic plants were adhesive and shriveled.

As in *Arabidopsis*, ectopic expression of cgo-miR159 led to the defective development of some tobacco pollen grains. The pollen grains of the wild-type were prolate and elliptical in the equatorial view and trifid round in the polar view (Figure 10a–c), whereas those of transgenic lines revealed more irregularly shaped pollen and were stuck to each other (Figure 10d–f). The pollen grains of cgo-miR159-overexpressing plants were often scattered in clumps but those of wild-type plants appeared granular. Therefore, it is reasonable to hypothesize that cgo-miR159 may alter the biophysical properties of pollen.

**Figure 11:**
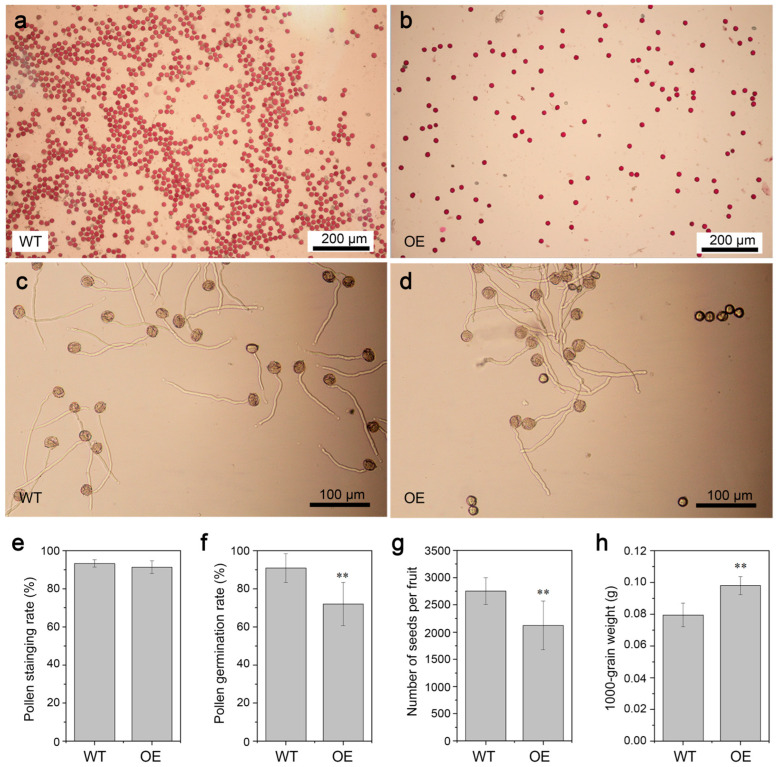
Phenotype changes in pollen viability of wild-type and cgo-miR159-overexpressed tobacco. (**a**,**b**) Observation of pollen grains with Alexander solution; (**c**,**d**) observation of in vitro pollen germination; (**e**) statistical results of pollen staining rate (*n* = 20 views); (**f**) statistical results of pollen germination rate (*n* = 12 views); (**g**) statistical results of seed number and weight (*n* = 12); (**h**) statistical results of seed weight. (*n* = 10). ** depicts a statistically extremely significant difference (*p* < 0.01).

Alexander staining (Figure 11a,b) and in vitro pollen tube germination assays (Figure 11c,d) were performed to examine the pollen viability. Statistical analysis shows no significant difference in the staining rate of mature pollen grains between the two tobacco genotypes (Figure 11e). However, cgo-miR159 overexpression significantly reduced pollen germination (Figure 11f). In contrast, the seed number per fruit of transgenic tobacco was lower than that of wild-type plants, whereas the thousand seed weight of transgenic lines was higher than that of wild-type plants (Figure 11g,h). These results demonstrate that cgo-miR159 overexpression reduces pollen fertility but may facilitate seed matter accumulation.

## 4. Discussion

miR159 has been identified as one of eight highly conserved miRNAs in plants and plays a role in many developmental and physiological processes across plant species [10,29]. Sequence conservation was observed in cgo-miR159 but the 3′ end was relatively poorly conserved (Figure 1c). Phylogenetic tree analysis shows that the miR159s from 21 species were randomly divided into monocot and dicot groups (Figure 1b). Interestingly, *cgo-MIR159*, *aof-MIR159*, and *vac-MIR159* were located at the junction of the two groups, which may explain why many characteristics of plants in the Orchidaceae, Liliaceae, and Bromeliaceae families fall between those of monocots and dicots. This result reflects their unique evolutionary history and may confer unique functional properties.

qPCR analysis reveals that, among different tissues of *C. goeringii*, the highest expression of miR159 was observed in the leaves during the reproductive growth stage (Figure 2c), suggesting a potential function of miR159 during leaf development. Among the floral organs, the activity of cgo-miR159 was highest in the stamens, suggesting that miR159 is also an important regulator of stamen and pollen development (Figure 2d). These predictions should be confirmed in subsequent studies.

MiR159 has been reported to participate in regulating the development of floral organs, particularly the stamen. For example, miR159 has been implicated in anther development by targeting the expression of *MYB33* and *MYB65* in *Arabidopsis* [8]. In yellow lupines (*Lupinus luteus*), GA3 controls *LlGAMYB* expression via a miR159-dependent pathway during late anther development [30]. In this study, overexpression of cgo-miR159 regulated early anther opening and pollen release in *Arabidopsis* and tobacco (Figure 4 and Figure 7). Similarly, after overexpression of cgo-miR159, the levels of *MYB33* and *MYB65* in *Arabidopsis* were significantly decreased (Figure S1e), and their sequences met the criteria for complementary pairing (Figure S2) [31]. The expression of these two genes also decreased in transgenic tobacco, but did not show significant difference from WT (Figure S1e). This may be due to inappropriate sampling time or tissue sampling (seedling leaves). Additional experiments on anther dehiscence in tobacco confirmed that transgenic anthers were easier to dehiscence than the wild-type anthers (Figure 8). In addition, when the anther of wild-type tobacco was completely dehiscent, the stigma was close to that of the four longer stamens (Figure 7d), which improved pollination probability. In contrast, the stigma of cgo-miR159 overexpressed tobacco was closer to that of short stamens. To compensate for this shortfall, the filaments of the longer stamens were bent downwards to make their anthers closer to the stigma of the transgenic lines (Figure 7h). This range of stamen behaviors may be a mechanism to compensate for pollen loss caused by early anther dehiscence. In contrast, the pistil was less affected by the ectopic expression of cgo-miR159, which led to slight premature atrophy of the pistil in *Arabidopsis* (Figure 6c).

In the mutant of *myb33*, *myb65*, two target genes of miR159, which result in defects in male meiotic cytokinesis, produce diploid pollen with defective pollen wall morphology [32]. However, the role of miR159 in the regulation of *GAMYB* during male development is unclear in most species [10]. As shown by microscopic observation, the *35S:cgo-MIR159* pollen of *Arabidopsis* and tobacco showed shrinkage and adhesion (Figure 6 and Figure 10). This is analogous to findings in *Brassica campestris* [19]. Interestingly, the Alexander staining rates of transgenic and wild-type pollen grains were similar but their pollen germination rates were significantly different (Figure 11). One reason for this is that the cell nucleus of some transgenic pollen grains is incomplete, leading to incomplete cell staining (Figure S3) [33]. Second, during the experimental process of pollen collection, partial pollen with developmental defects was aspirated from the supernatant owing to its light weight; therefore, it could not be included in the statistical results. Previous studies indicate that the tapetum plays a vital secretory role in the production of functional pollen grains, providing nutrients for pollen development and pollen wall components [34,35]. The deposited callose is degraded by callase enzymes secreted by tapetal cells when the tetrads mature into pollen grains, and a male-specific gene, *MYB35*, acts in tapetal cells at an early stage of flower development [36]. Therefore, the abnormal development of tapetosomes may have caused the aberrant pollen in cgo-miR159-overexpressing anthers observed in this study. However, further experimental validation is required to confirm this prediction.

Fruit yield and quality were significantly constrained by the negative effects of malformed pollen. In cgo-miR159 transgenic plants, ovule abortion was observed, and seed yield was reduced (Figure 3 and Figure 11). The same phenomenon has been observed in other plants, such as *Arabidopsis mir159ab* with small and misshapen seeds, rice *STTM159* with small grains, and *SlMIR159*-overexpressing tomatoes with precocious fruit initiation and seedless fruits [17,20,37]. These effects are likely caused by a combination of factors, like blocked pollen germination and reduced pollen–stigma interaction. Interestingly, the relative weight of transgenic seeds was significantly higher than that of seeds from wild-type plants. This indicates that cgo-miR159 may be beneficial for seed substance accumulation during seed development or may be involved in the self-compensation of plants in response to transgenic adversity. Specific explanations for these phenomena and speculations need to be explored in future experiments.

## 5. Conclusions

In summary, cgo-miR159 plays a crucial role in the development of reproductive organs in plants. miR159 was highly expressed in the reproductive petals and anthers of *C. goeringii*, with peak expression occurring when the flower bud reaches approximately 1 cm, suggesting its key role at this stage of leaf and anther development. Transgenic experiments in model plants show that overexpression of cgo-miR159 led to premature anther division and abnormal pollen morphology, including shriveling, stickiness, and incomplete nuclear structure, which ultimately affected fruit development. These findings confirmed that miR159 was involved in regulating pollen and anther development, consistent with its known function in other species like *Arabidopsis*. Additionally, reduced seed yield and ovule abortion in transgenic plants further supported the idea that cgo-miR159 significantly influenced fruit and seed development. This research provides new insights into the role of miR159 in orchid reproductive biology, highlighting its importance in flower organ development and fertility regulation. Moreover, this research offers valuable references for the genetic improvement of *Cymbidium* and other orchids, which could have broader implications for enhancing the ornamental and economic value of orchids. The study also contributes to a deeper understanding of the regulatory role of miRNAs in plant reproduction, and its potential applications in orchid breeding, plant biology, and agricultural practices could have a global impact.

## Data Availability

All data generated or analyzed during this study are included in this published article and its supplementary information files. The sequences described here are available in the supplemental files and at Genbank: OP856784.

## Supplementary Materials

The following supporting information can be downloaded at: https://www.mdpi.com/article/10.3390/genes16010035/s1, Figure S1: PCR analysis of cgo-*MIR159* gene fragment amplified from transgenic plants; Figure S2: Comparison results of cgo-miR159 with its heterogenic target genes in RNAhybrid; Figure S3: The incomplete nuclei of pollen grains affect the staining situation; Table S1: Sequences of primer and use; Table S2: The list of cgo-miR159 sequences
