# Supplementary figures and images for: Overexpression of Cymbidium goeringii Cgo-miR159 Regulates Anther Dehiscence and Pollen Development in Arabidopsis and Tobacco
Source: Genes (Basel). 2024 Dec 29;16(1):35. doi: 10.3390/genes16010035 (PMC11765276; doi:10.3390/genes16010035)

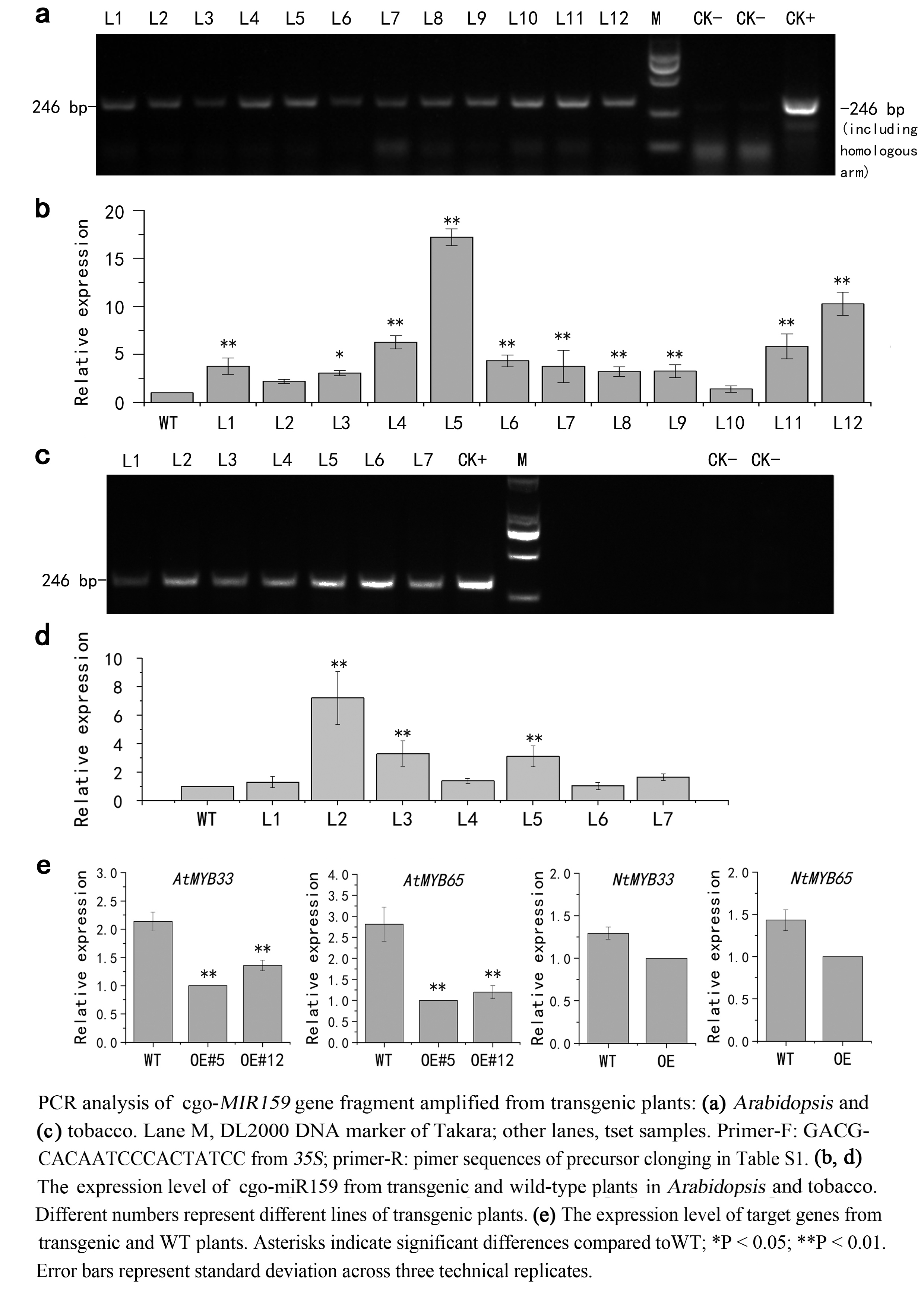

Supplement: Supplementary file 1 [file genes-16-00035-s001.zip › Fig. S1.jpg]

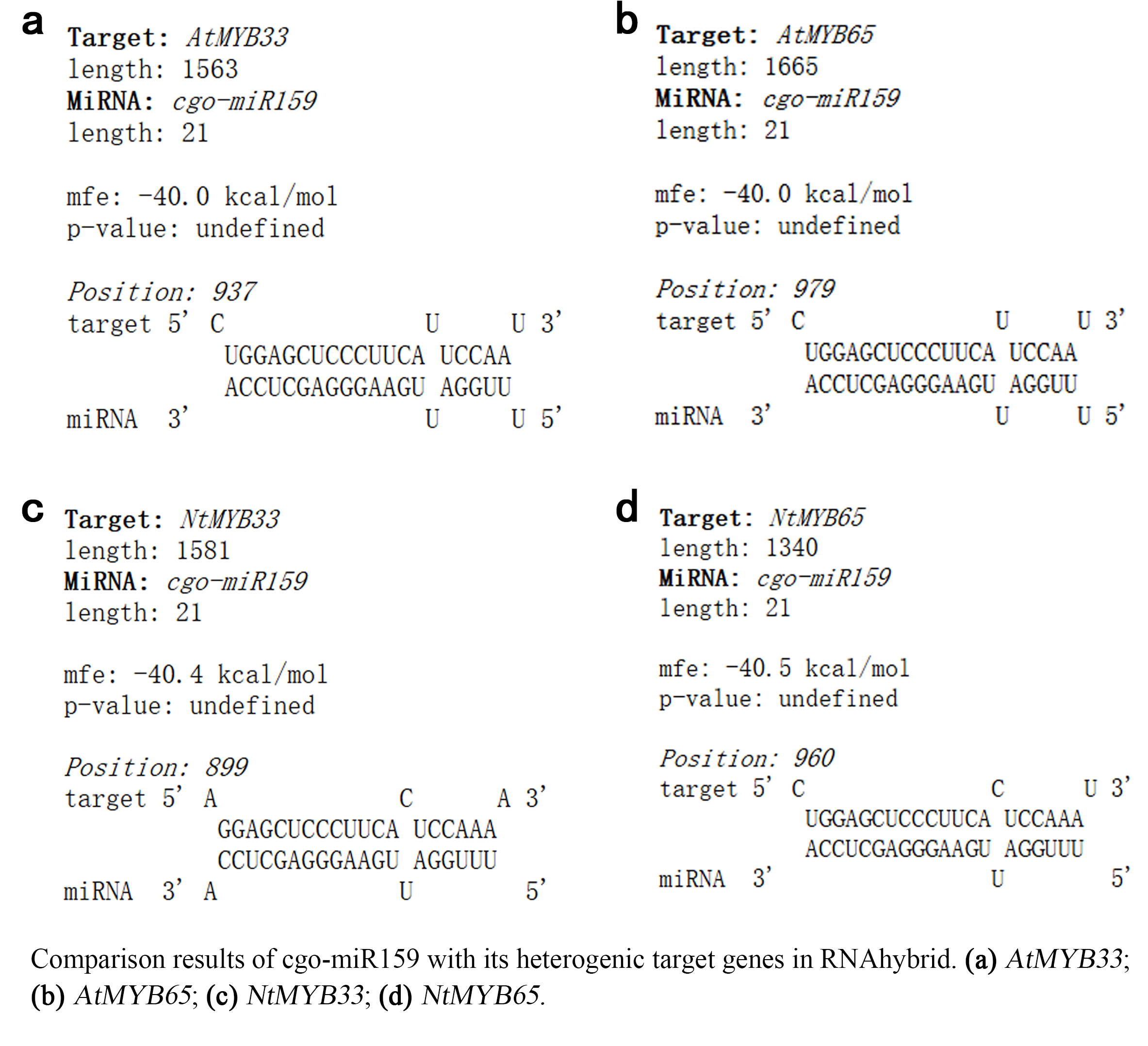

Supplement: Supplementary file 1 [file genes-16-00035-s001.zip › Fig. S2.jpg]

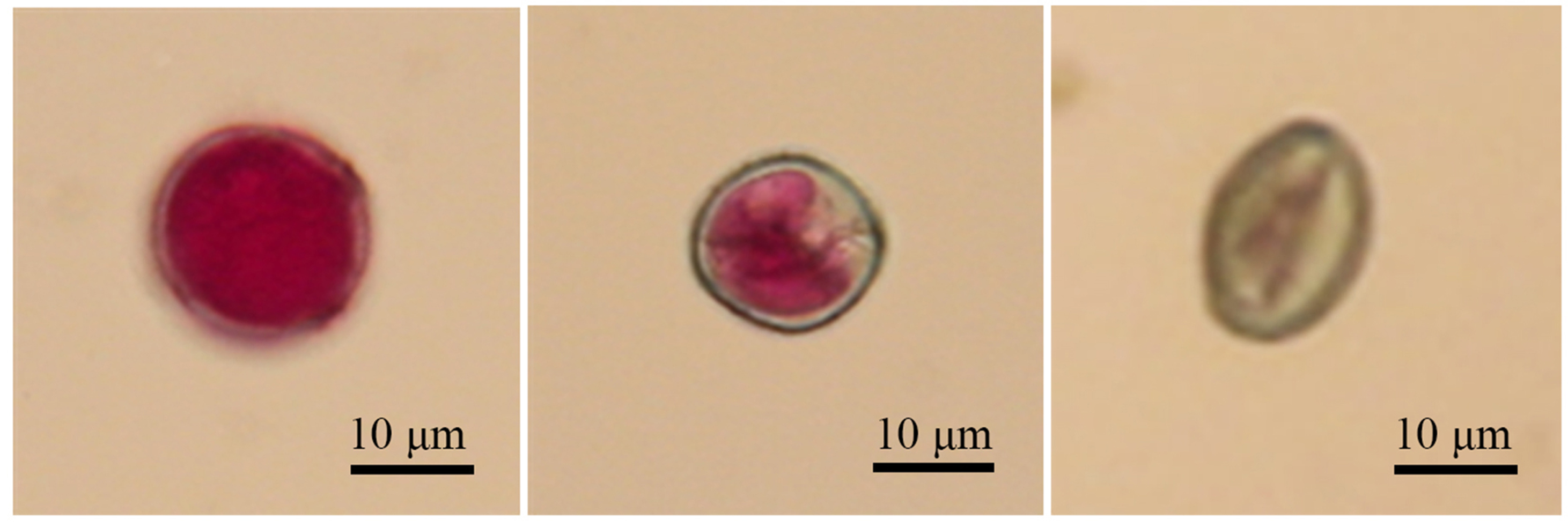

Supplement: Supplementary file 1 [file genes-16-00035-s001.zip › Fig. S3.jpg]
